# Frequency of Retinopathy in Patients Newly Diagnosed With Type 2 Diabetes Mellitus

**DOI:** 10.7759/cureus.36513

**Published:** 2023-03-22

**Authors:** Wagmah Javed Khan, Tahir Aslam

**Affiliations:** 1 Internal Medicine, Hayatabad Medical Complex Peshawar, Peshawar, PAK

**Keywords:** type 2 diabetic retinopathy, insulin, hemoglobin a1c, diabetes mellitus, retinopathy

## Abstract

Background: The most prevalent microvascular consequence of diabetes mellitus on the eye is diabetic retinopathy, which is also one of the major reasons for poor vision in the working-age population. The objective of this study was to estimate the prevalence of type 2 diabetic retinopathy in participants.

Methods: The Department of Medicine at the Hayatabad Medical Complex in Peshawar performed a six-month cross-sectional study from May to November 2022. A total of 196 patients with type 2 diabetes mellitus were included in the research.

Results: Ages ranged from 18 to 60 years with a mean age of 37.59 ± 10.21 years, with the majority of the patients (n=16) belonging to the fourth decade. Thirty-one individuals (15.81%) with clinically diagnosed type 2 diabetes mellitus had diabetic retinopathy, of which 12 (6.12%) were females and 19 (9.69%) were males. Among patients with diabetic retinopathy, glycated haemoglobin (HbA1C) was determined to be 9.4 ± 1.5, and among those with other types of retinopathy, background retinopathy was detected in 11 (5.61%) men and seven (3.57%) female patients.

Conclusion: The majority of diabetic retinopathy patients in the current study were older than 40 years and were primarily males. In newly diagnosed type 2 diabetes mellitus participants, retinopathy occurred in 15.81% of cases (n=31), with background retinopathy accounting for the majority of cases (n=18, 9.18%).

## Introduction

Diabetes mellitus (DM) is caused by higher levels of blood glucose due to the lack of production of insulin by the body, resistance to insulin, or both. Over 451 million people have diabetes globally, and according to the second National Diabetes Survey of Pakistan and the International Diabetes Federation, the prevalence of diabetes is over 26% in Pakistan. Significant socioeconomic transition is predicted to lead to an increase in the proportion of diabetes patients in the upcoming years [1-5].

Blood glucose levels that are consistently high lead to widespread vascular damage and a variety of both micro and macrovascular problems. A brief microvascular consequence of diabetes in the eye is diabetic retinopathy (DR). DR will proceed from its minor anomalies to its severe forms if early identification and therapy are not provided. Tractional retinal detachment, macular oedema, and neovascular glaucoma worsen DR and finally cause seriously impaired vision [6-8]. According to estimates, 27.0% of people with DM worldwide have DR. According to a pooled study of many hospital-based researches, Pakistan has a 28.2% frequency of DR. The World Health Organization (WHO) estimates that DR accounts for 37 million blind reported cases. The treatment of diabetic comorbidity is difficult due to visual loss brought on by DR, which also reduces life span and lowers the standard of living [9-12]. The variables most consistently linked to the development of DR in diabetic patients include increased fasting blood sugar levels, prolonged duration of diabetes, obesity, hypertension, being on insulin therapy exclusively, history of diabetes in relatives, and low socioeconomic position [13,14].

Healthy blood pressure management, important early detection, managing hypertension, and frequent follow-up in a diabetic ophthalmology clinic can all help to lower the threat of DR to vision. Treatment options for DR include prompt laser therapy, antivascular endothelial growth factor medications, steroid intraocular injections, and intraocular surgeries. In affluent countries, the risk and epidemiological components of DR have been described and evaluated, and a small number of studies have been performed in poorer countries as well [15,16]. Therefore, The objective of this research was to estimate the prevalence of type 2 DR in participants.

## Materials and methods

A six-month cross-sectional study from May to November 2022 was carried out in the Department of Medicine, Hayatabad Medical Complex, Peshawar, after the approval by the Research Evaluation Unit, College of Physicians and Surgeons Pakistan (approval number: CPSP/REU/MED-2017-21-12822), permission by the hospital ethics council, and written informed consent from the participants.

Participants in the study were between the ages of 18 and 60 and had been diagnosed with type 2 DM. The research excluded patients who had retinal artery occlusion, hypertension, retinal vein occlusion, and sickle cell retinopathy. Values of 200 mg/dl or higher on two consecutive fasting blood sugar, randomized blood sugar, and glycosylated haemoglobin (HBA1c) tests are considered diagnostic conditions of type 2 diabetes, according to WHO criteria released in 1999 and revised in 2007 [17].

After treating the eye with 1.0% tropicamide eye drop, skilled optometrists used a 90-diopter Volk lens and slit lamp bio-microscope to determine the presence of DR. The three ophthalmologists who were double-masked for their observations inspected each eye. A professional ophthalmologist was contacted in cases of disagreement to arrive at a consensus diagnostic of DR.

IBM SPSS Statistics for Windows, Version 23.0 (Released 2015; IBM Corp., Armonk, New York, United States) was used for statistical analysis. Descriptive statistical analysis was done on the patient's basic information (gender, age), and a chi-square test and a paired t-test were done on their qualitative information (prevalence of DM, with and without DR).

## Results

The study took six months to complete, from May to November 2022. A total of 196 patients who fulfilled the requirements were included. Ages ranged from 18 to 60; the bulk of patients (n=116) were mostly in their fourth decade and had a mean age of 37.59 ± 10.21 years. Of the patients, 63.26% were males (n=124) and 36.74% were females (n=72) (Table 1).

**Table 1:** Age and gender distributions of the patients

Variables	No. of Patients	Percentage
Gender Distribution		
Male	124	63.26
Female	72	36.74
Age in Years		
18-40	17	8.67
41-50	116	59.19
51-60	63	32.14

DR was seen in 31 (15.81%) participants with type 2 DM. Of these, 12 (6.12%) were females and 19 (9.69%) were males. Also, among the 31 patients who had DR, the majority (n = 14) were in the age group of 41-50 years, followed by the age groups of 51-60 years (n = 12) and 18-40 years (n = 5). The cohort gap was not statistically significant (p-value = 0.8459). Those with DR had an HbA1C (%) of 9.4 ± 1.5, whereas those without DR had 7.3±2.4 (Table 2).

**Table 2:** Distribution of DR patients by gender, age, and diagnostic criteria DR: diabetic retinopathy; HbA1C: glycated haemoglobin

Variables	No. of Patients	Percentage	P-Value
Gender			
Female	12	6.12	
Male	19	9.69	
Total	31	15.81	
DR patients by age			0.8459
18-40 Years	5	29.41	
41-50 Years	14	12.06	
51-60 Years	12	19.04	
Diagnostic criteria			
Parameter	With DR	Without DR	
Fasting plasma glucose (mg/dl)	219 ± 36.5	142.8 ± 38.5	
HbA1C	9.4 ± 1.5	± 2.4	

Background retinopathy was discovered in 11 patients (5.61%) who were males and seven (3.57%) who were females. Preproliferative and proliferative retinopathy was discovered in five and three (2.55%; 1.53%) males, respectively, and three and two (1.53%; 1.02%) females, respectively (Table 3).

**Table 3:** Several DR types in the research group DR: diabetic retinopathy

DR types	No. of Patients	Percentage
Background Retinopathy		
Male	11	5.61
Female	7	3.57
Preproliferative Retinopathy		
Male	5	2.55
Female	3	1.53
Proliferative Retinopathy		
Male	3	1.53
Female	2	1.02

## Discussion

The most prevalent endocrine metabolic condition is DM. In the current research, DR was found in 31 (15.81%) patients with type 2 DM. There were 12 females (6.12%) and 19 males (9.69%) among them. HbA1C was found to be 9.4±1.5 in patients with DR. In participants with other types of retinopathy, background retinopathy was found in 11 (5.61%) males and 7 (3.57%) females. Different diagnostic criteria make it difficult to determine the actual frequency of DM patients; however, several studies have indicated that it is between 6% and 9% in Pakistan [18]. It was believed that 13% of diabetes patients in Pakistan have DR, although other sources have recorded rates of 14-18.9%. DR is a significant contributor to blindness in people with type 2 DM. It is estimated that more than 200 million people will be affected by DR in 2040 [19,20].

We performed a prospective study among type 2 DM patients who had just received a diagnosis, and we discovered that 15.81% of the individuals had DR. In a study conducted in the southern regions of Pakistan, DR was present in 12% of recently diagnosed diabetic patients [21]. Corresponding to this, a study from India claimed that this number was 11.2%, while a study from the United Kingdom showed that the percentage of people with diagnosed retinopathy there was 18% [22,23]. These variances may result from racial diversity, gender disparities, and age-group presentations. This may be shown by contrasting our findings with those of a study done similarly in Abbottabad [24]. The frequency was determined to be 16% in that study, which had an average age of 47.12 ± 3.2 years and was predominately female, compared to an average age of 37.59 ± 10.21 years in the current study and a predominately male population. We discovered that background retinopathy made up the majority of cases (9.18%), followed by pre-proliferative (4.08%) and proliferative (2.55%). These findings are roughly equivalent to those of Hayat et al. [24].

HbA1C (%) in our research group was 9.4 ± 1.5, and the fasting plasma sugar value in the participants with DR was 219 ± 36.5. Our results strengthen the relationship between HbA1c and fasting glucose concentration in retinopathy patients that was previously postulated by Rema et al. [22] and Abdollahi et al [25]. The degree of retinopathy is correlated with HbA1c levels and systolic blood pressure.

Limitations

The study was limited to patient examinations at the Hayatabad Medical Complex in Peshawar. Only consenting participants with the requisite data were included in this study. Patients with retinal artery occlusion, hypertension, retinal vein occlusion, and sickle cell retinopathy were precluded from the study. Due to the limited number of diabetic participants, it is conceivable that the estimation does not accurately reflect the prevalence of DR.

## Conclusions

The majority of DR patients were over the age of 40 and were predominantly men. Participants with newly diagnosed type 2 DM exhibited DR in 31 cases (15.81%), with background retinopathy comprising the majority of cases (n=18; 9.18%).
